# Higher variability of bacterial communities across space than over time in Antarctic lakes, and contrasting assembly processes

**DOI:** 10.1128/aem.01079-25

**Published:** 2025-10-22

**Authors:** Diego Ahumada, Guillaume Schwob, Magdalena Osorio, Maria Soledad Astorga, Céline Lavergne, Nazli Olgun, Frederic Thalasso, Elie Poulin, Julieta Orlando, Léa Cabrol

**Affiliations:** 1 Laboratory of Molecular Ecology, Department of Ecological Sciences, Faculty of Sciences, University of Chile 14655, Santiago, Chile; 2 Millennium Institute Biodiversity of Antarctic and Subantarctic Ecosystems (BASE) 676466 , Santiago, Chile; 3 Universite Claude Bernard Lyon 1, UMR Ecologie Microbienne, CNRS 5557, INRAE 1418 27098, Marcy L’Etoile, France; 4 Laboratory of Microbial Ecology, Department of Ecological Sciences, Faculty of Sciences, Universidad de Chile 14655, Santiago, Chile; 5 Departamento de Ciencias y Recursos Naturales, Universidad de Magallanes 28062, Punta Arenas, Chile; 6 Departament de Biologia Marina i Oceanografia, Institut de Ciències del Mar (ICM-CSIC) 58341 , Barcelona, Spain; 7 HUB Ambiental UPLA, Departamento Ciencias y Geografía, Facultad de Ciencias Naturales y Exactas Universidad de Playa Ancha, Valparaiso, Chile; 8 Eurasia Institute of Earth Sciences, Istanbul Technical University 52971, Istanbul, Turkey; 9 Departamento de Biotecnología y Bioingeniería, Centro de Investigación y de Estudios Avanzados del Instituto Politécnico Nacional (Cinvestav), Mexico City, Mexico; 10 Aix-Marseille University, Univ Toulon, CNRS, IRD, Mediterranean Institute of Oceanography (M.I.O.) UM 110 128791, Marseille, France; Colorado School of Mines, Golden, Colorado, USA

**Keywords:** lake, assembly processes, Antarctic South Shetland islands, temporal dynamics, microbial ecology, variability

## Abstract

As sentinels of environmental changes, Antarctic lakes are ideal systems for studying the temporal and spatial dynamics of microbial communities. However, the relative magnitude and underlying mechanisms driving these variations remain poorly understood. Studying the spatiotemporal variation of microbial communities is crucial to provide a robust baseline for predicting ecosystem responses to global changes. Here, we investigated the spatial and inter-annual variation of bacterial community structure and their underlying assembly processes across sediment and water habitats in 11 lakes on the Fildes Peninsula, Maritime Antarctica, sampled during austral summers from 2017 to 2023, using 16S rRNA gene sequencing. The communities primarily clustered by habitat, with higher diversity in sediment (characterized by *Rhodoferax, Intraporangiaceae,* and *Vicinamibacterales*) compared to water (characterized by *Polaromonas*, *Flavovacterium*, and *Sporichthyaceae*). Spatial turnover of communities dominated over inter-annual variation in both habitats. Accordingly, the temporal core microbiome showed greater stability than the spatial core. The conserved bacterial communities (core communities) over time and across space exhibited a strikingly similar taxonomic composition. Community assembly processes differed between habitats, with a stronger contribution of dispersal limitation in sediment, versus ecological drift in water, as expected from the differences in connectivity within each habitat. Spatial and temporal variations in sediment were driven by globally similar assembly processes. In contrast, in water communities, different assembly processes explained the spatial and temporal variation. These insights emphasize the need to consider both spatial and temporal scales and various habitat types when predicting future bacterial dynamics in Antarctic lakes in a changing environment.

**IMPORTANCE:**

Understanding the inherent baseline microbial dynamics in Antarctic lakes is crucial for predicting their responses to environmental changes. Our findings underscore the predominance of spatial (rather than inter-annual) factors in shaping bacterial communities and highlight the slightly higher contribution of stochastic processes in sediment compared to water habitats. The stochastic processes differed considerably among habitats. The greater stability of the temporal core microbiome suggests a certain degree of resilience toward possible seasonal fluctuations between the inter-annual sampling dates. In water, dispersal limitation and homogeneous selection played a greater role in the spatial than in the temporal turnover of communities, whereas environmental filtering exerted a stronger influence over time. Future studies should integrate both spatial and temporal dimensions in evaluating microbial community variability to improve forecasting of ecosystem shifts in response to global change and thus provide a better baseline for Antarctic biodiversity conservation and management.

## INTRODUCTION

Maritime Antarctica has been undergoing rapid climatic changes, including rising temperatures, altered precipitation patterns, and changes in ice cover (1–3). Lake ecosystems are recognized as effective early indicators (“sentinels”) of environmental changes, such as those induced by climate warming, due to their sensitivity and rapid response (4, 5). Microorganisms inhabiting Antarctic lakes play a crucial role in globally relevant biogeochemical cycles such as the carbon and methane cycles (6–9). Current environmental shifts are expected to impact lake microbial community structures and their ecological roles in the long term (10, 11). However, independent of the long-term effect of global changes, lacustrine communities display intrinsic temporal and spatial variability at shorter time scales and local scales, which remains largely understudied. The lack of temporal studies and long-term monitoring has been identified as a gap in microbial ecology, especially in Antarctica, preventing a comprehensive understanding of biodiversity changes over time and/or in response to external stressors (12, 13). It is therefore critical to understand the spatial and temporal variation of microbial communities at mid-term (5–10 years) and at a local scale (1–10 km), along with their underlying assembly processes (10), to provide a robust baseline for predicting ecosystem responses to global changes.

Microbial communities are shaped by both deterministic and stochastic assembly processes (14–19). Deterministic processes arise from selection pressure imposed by abiotic or biotic factors (20), leading communities to either converge under similar environmental conditions (homogeneous selection) or diverge under distinct environmental conditions (variable selection) (21, 22). Stochastic processes involve ecological drift (i.e., stochastic changes in population sizes) and dispersal, the latter leading either to community convergence through frequent species exchanges (homogeneous dispersal) or to community divergence by limiting species exchange (dispersal limitation) (16, 21, 22). Multiple factors, including environmental heterogeneity, habitat specificity, dispersal ability, spatial scale, functional guild, and taxonomic resolution, could influence the relative contribution of different community assembly processes (23–29). However, a key challenge remains in disentangling how different assembly processes shape the temporal and spatial variation of microbial community.

In lake ecosystems, the temporal variability of bacterial communities is mainly linked to seasonal events, such as water column mixing and climatic trends (30, 31). Seasonal shifts in the relative importance of microbial assembly processes, particularly the deterministic ones, have been identified in lakes (32, 33). However, most studies have focused exclusively on the intra-annual effects of seasons on microbial assembly processes in a limited number of lakes (32–36), while very few studies have considered the inter-annual variability of lake communities during the same season (37, 38). Aguilar and Sommaruga (37) found that homogeneous selection (i.e., a deterministic process) was the main assembly process explaining the variation of lake bacterial communities on a 2-year inter-annual time scale. In addition, the investigation of spatiotemporal dynamics and assembly patterns in lakes usually focuses on the water habitat (37–44), overlooking the sediment. Microbial dynamics are expected to differ between water and sediment given the strong influence of physicochemical habitat properties on microbial assembly patterns, as well as the limited exchanges between both habitats (45–47). In Antarctic wetlands, dispersal limitation and drift were hypothesized to generate microdiversification leading to niche differentiation across lentic, lotic, and terrestrial habitats even at a sub-taxa level (29, 48). Here, we consider a system of highly connected shallow lakes in a restricted area exposed to the same climatic and geological context, offering the opportunity to study two contrasting habitats, water and sediment, each with unique intrinsic environmental conditions and microbial community composition (49).

In this study, we aim to: (i) assess and hierarchize the magnitude of spatial and temporal (inter-annual) variations of bacterial communities across 11 Antarctic lakes over a 6-year period, based on four sampling campaigns conducted during the austral summer; and (ii) elucidate the ecological assembly processes underlying the spatial and temporal turnover of these communities in contrasting habitats (water and sediment).

## MATERIALS AND METHODS

### Sample collection and processing

**Fig 1:**
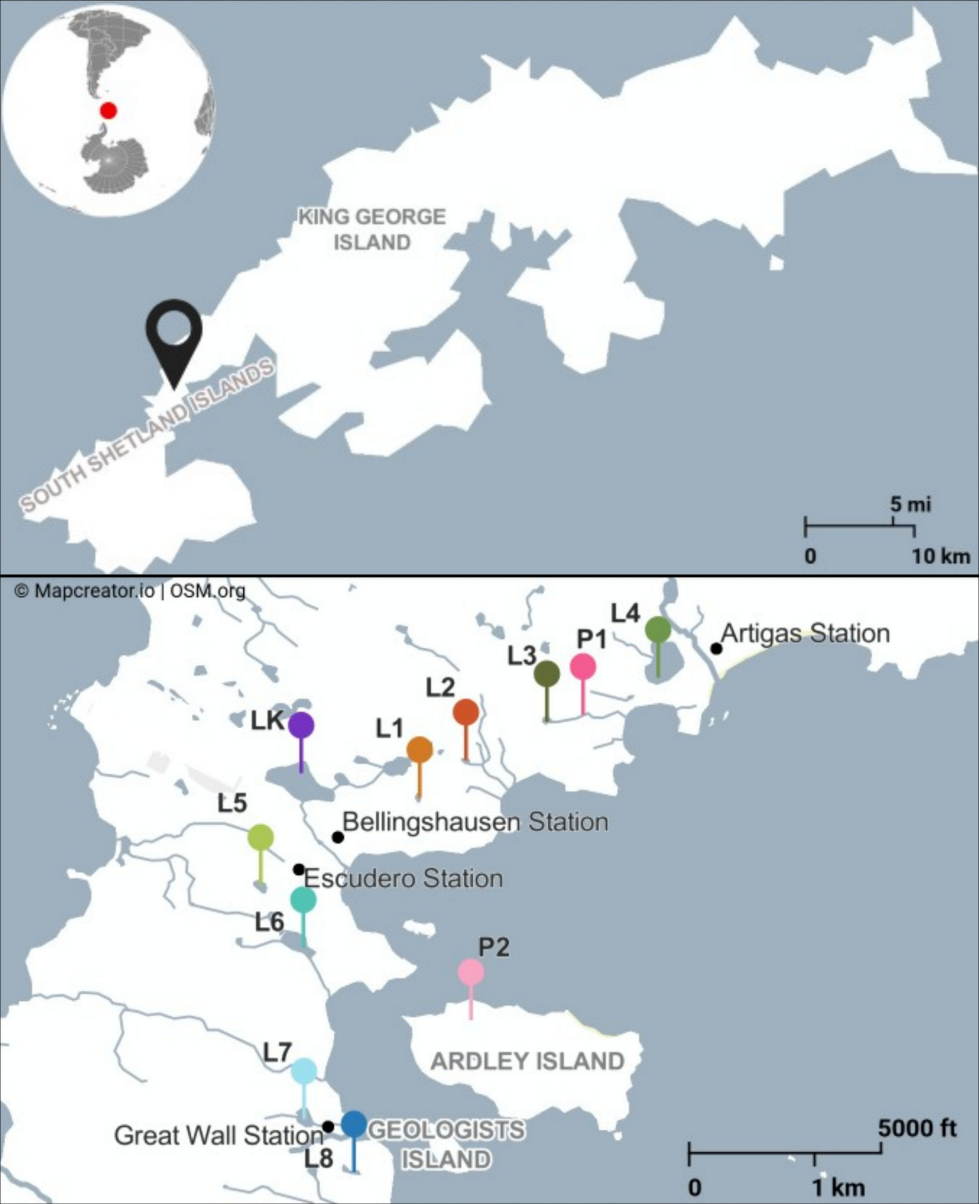
Sampled ecosystems. The top panel shows King George Island, part of the South Shetland Islands, Maritime Antarctica, and the label indicates Fildes Peninsula. The lower panel shows the 11 lakes sampled in the Fildes Peninsula and Ardley Island in the frame of this study.

A set of 165 water and sediment samples was collected from 11 lakes (including two shallow ponds) located in Fildes Peninsula on King George Island, Maritime Antarctica (Table S1; Fig. 1). The same lakes were sampled over four different years (2017, 2019, 2021, and 2023) from December to early March, all during the austral summer. The sampling periods vary from 3 to 73 days among different years (Fig. S1A).

At each lake, surface water (50 cm depth) and underlying superficial sediment (0–10 cm depth) were sampled from the littoral zone (i.e., 2 m from the shore) at the same point. In addition, given the large size of Lake Kitiesh (LK), samples were exceptionally collected from several locations across the lake, including the central, eastern, western, northern, and southern zones, and at different depths (surface/bottom), according to the details given in Table S2. All samples were collected in duplicates (points A and B, at some meters of distance from each other) unless stated otherwise in Table S1. Sediment and water samples were stored in sterile Ziploc bags and 5 L containers, respectively, and maintained at 4°C until further processing. Following the procedure of Cabrol et al. (50), water samples ranging from 1,000 to 2,000 mL were directly filtered on 0.22 µm cellulose ester filters (MF-Milipore) as soon as possible (< 24 h) after sampling to obtain the bulk community. The resulting filters were placed in sterile 5 mL Eppendorf tubes and stored at −20°C. Sediment aliquots were homogenized and transferred into 2 mL sterile cryotubes and stored at −20°C.

### Physicochemical variables

Dissolved oxygen, temperature, pH, turbidity, conductivity, and redox potential were measured *in situ* in the lake water using a multiparameter probe (HI 9828, Hanna Instrument, Mexico). The proportion of particulate organic matter (relative to the total suspended solids TSS) in water samples was determined by filtering onto pre-weighted combusted GF/F grade glass microfiber filters (0.7 µm pore size, Whatman), drying overnight at 105°C to calculate the TSS, and incinerating at 550°C for 2 h (51).

Climatological data were obtained from records of the Eduardo Frei Montalva Station, located on Fildes Peninsula near the sampling sites (https://climatologia.meteochile.gob.cl/). The average monthly air temperature between 2016 and 2023 (spanning the four sampling periods) is shown in Fig. S1B. The 11 lakes have been previously characterized in terms of carbon budget (8), lithology, and biogeochemistry (52).

### DNA extraction and sequencing

Genomic DNA was extracted from water and sediment samples using the DNeasy PowerWater Kit and PowerSoil Pro Kit (Qiagen), respectively, following the manufacturer’s recommendations. The concentration and quality of the extracted DNA were assessed using a NanoDrop spectrophotometer (Thermo Scientific NanoDrop Lite Plus Spectrophotometers). The V4-V5 hypervariable region of the 16S rRNA gene was amplified using the primers 515F (5′-GTGYCAGCMGCCGCGGTA-3′) and 928R (5′-CCCCGYCAATTCMTTTRAGT-3′) (53). The amplicons were sequenced using the paired-end sequencing technology (2 × 250 bp) on the Illumina MiSeq platform at the UWBC DNA Sequencing Facility (University of Wisconsin-Madison, USA).

### Sequencing data processing

The 16S rRNA gene forward and reverse reads were merged utilizing the Fast Length Adjustment of Short Reads (FLASH) software (min overlap = 10 nt, max mismatch density = 0.1) (54). The merged reads were processed through Mothur v1.44.0 (55). Briefly, primers were removed, reads were trimmed based on their length (371–378 nt), and sequences with ambiguous nucleotides and >8 homopolymers were removed. Chimeric sequences were eliminated using UCHIME integrated into mothur (56). The reads were clustered into operational taxonomic units (OTUs) at a 97% identity threshold, and a relative abundance filter of 0.005% was applied (57). The taxonomic classification of the OTUs was performed using the *classify.otu* function against the SILVA database v138.1 (58). The number of sequences before and after pre-processing is summarized in Table S3. A total of 6,736,013 raw reads were generated from 165 sediment and water samples. Once processed, 6,053,244 cleaned sequences were obtained and distributed into 1,451 OTUs (412 ± 96 OTUs per sample on average). The OTU table was rarefied to 7,690 reads per sample after eliminating two samples with low abundance (L1-A sediment 2021 and LK-B sediment 2017). Rarefaction curves are shown in Fig. S2.

### Alpha- and beta-diversity analysis

The Shannon index and OTU richness were calculated using the e*stimate_richness* function from the rarefied OTU table. Significant variations of alpha diversity indices between compartments were evaluated using the Wilcoxon test. For beta-diversity analysis, OTU counts were subjected to Hellinger transformation from the rarefied OTU table before calculating Bray–Curtis dissimilarity distances. Distance matrices were ordinated by a principal coordinate analysis (PCoA) using the *plot_ordination* function implemented in the phyloseq package (59). The contribution of compartment (i.e., sediment/water), ecosystem (i.e., identity of lake), and sampling year in explaining the differences in bacterial community composition was assessed by permutational multivariate analysis of variance (PERMANOVA) using the *adonis2* function in the vegan package (60). The community variability within each habitat was evaluated by analysis of multivariate homogeneity of group dispersion (*betadisper* function, vegan package).

For each habitat, variation partitioning analysis (VPA) was conducted with the *varpart* function in the vegan package (60) to examine the relative contribution of spatial, environmental, and temporal factors in differentiating bacterial communities. For the spatial factor, spatial distance between samples was calculated from the longitude and latitude coordinates of each sampling location using the *earth.dist* function in the fossil package (61). The resulting distance matrix was transformed into spatial predictors using the principal coordinates of a neighborhood matrix (PCNM) approach (62). The 13 most significant PCNM variables were forward-selected following the approach described by Zhang et al. (63) and used as spatial matrix. For this, all geographic PCNM variables were first used as input of a db-RDA model (*capscale* function) to explain the microbial community structure (Hellinger-transformed rarefied OTU table). Second, their significant contribution to the db-RDA model was tested using the *ordi2step* function, selecting the variables with *P*-value < 0.05. Finally, these variables were tested with the *vif.cca* function in order to select the variables with variance inflation factor (VIF) below 10. For temporal variables, the time elapsed between sampling dates (i.e., delta days) was used to perform a PCA, from which the first two principal components were extracted and used as temporal predictors in the VPA. To handle missing physicochemical measurements, the environmental variables were first imputed using the *imputePCA* function implemented in the missMDA package (64). The environmental variables were then normalized (centered and scaled) using the *decostand* function in VEGAN. The most relevant environmental variables were identified by forward selection following the same protocol used for PCNM variables to avoid overestimating explained variance and to mitigate multicollinearity between variables (63). The selected environmental variables were then used to conduct a PCA (*prcomp* function), and the five resulting principal component vectors were included in the VPA. The significance of partitioned fractions was tested by a permutation test for distance-based redundancy analysis using the *anova.cca* in the vegan package.

The OTUs differentially enriched between sediment and water compartments were identified through a linear discriminant analysis (LDA), with an LDA score threshold of 1.5 and *P*-value < 0.05, using the *run_lefse* function in the microbiomeMarker R package (65). The 20 highest-scoring OTUs from each compartment (Table S4) were visualized in a heat tree using the *heat_tree* function (MetacodeR package [66]), showing the log2 ratio of their median proportions between compartments and their relative abundance with respect to the total data set (summed per taxon when they shared the same taxonomic affiliation). Within each compartment, the relative abundance of the 10 highest-scoring OTUs was also visualized in a heatmap using the *pheatmap* function in the pheatmap R package (67).

The core microbiome was defined as the OTUs present in at least 75% of the samples, with a relative abundance higher than 0.1% per sample. The spatial core was defined by identifying the OTUs common to at least 75% of the different lakes for a given sampling year. The temporal core was defined by identifying the OTUs common to 75% of the sampling dates for a given lake. The relative abundances of OTUs from each spatial core of a given sampling year and each temporal core of a given lake were extracted to assess taxonomic composition. The spatial and temporal cores were computed independently in the sediment and water habitats using the *core_members* function in the microbiome R package.

### Ecological assembly process estimation

To quantify the relative contribution of stochastic and deterministic processes on bacterial community assembly, we applied the analytical framework described by Stegen et al. (22). As a prerequisite for applying this framework, we assessed the phylogenetic signal using Mantel correlograms (*mantel.correlog* function, vegan package), based on Pearson correlation coefficients between differences in taxa environmental optima and their phylogenetic distances, as described in Quiroga et al. (29). Environmental optima for abundant taxa (i.e., relative abundance >1% in each sample) were estimated by canonical correspondence analysis (*cca* function, vegan package) with the same explanatory variables as in the previous analysis (selected by forward selection). The global significance of the CCA model was tested using permutation tests (999 permutations), and the significance of individual axes was evaluated using separate permutation tests. OTU scores along the first significant CCA axis (*P* < 0.05) were extracted and used to calculate Euclidean distances between OTUs, which represent differences in environmental optima, or niche preference. Phylogenetic distances were computed from a rooted maximum-likelihood phylogenetic tree (constructed with PhyML v3.0 [68], with parameters ‘–-model GTR -f e -nclasses 4–search SPR -t e’) using the *cophenetic.phy* function in the ape package (69). Significance tests for each of the 30 phylogenetic distance classes were based on 999 permutations and a progressive Bonferroni correction. We obtained significant positive correlations in the lowest distance classes (Fig. S3), which is interpreted as evidence of a phylogenetic signal, thus allowing the application of Stegen’s framework.

The determination of community assembly processes relies on comparing the phylogenetic turnover between communities across samples (β mean nearest-taxon distance, βMNTD) to a null distribution of βMNTD. The phylogenetic trees required for the βMNTD matrix were generated as described above for the correlograms. The difference between measured βMNTD and the mean of the null distribution, expressed in units of standard deviations, is denoted as the β-nearest taxon index (βNTI). The obtained values of βNTI indicate that all pairwise OTU comparisons between two communities are more (when βNTI < −2) or less (when βNTI > +2) phylogenetically related than expected by chance, thus suggesting that communities are governed by homogenizing or variable selection, respectively (both being deterministic processes) (22). βNTI values range from −2 to +2 point to stochastic processes. These stochastic processes were further identified and quantified using the pairwise Bray–Curtis-based Raup-Crick dissimilarity index (RCBray) among samples (70). RCBray values between −0.95 and +0.95 indicate ecological drift. RCBray values lower than −0.95 reflect greater taxonomic similarity between communities than expected by chance, indicating that community turnover is driven by homogenizing dispersal. RCBray values higher than +0.95 indicate lower taxonomic similarity than expected by chance, suggesting that community turnover is driven by dispersal limitation (71).

The respective contributions of the five assembly processes (variable selection, homogeneous selection, dispersal limitation, ecological drift, and homogenizing dispersal) were calculated independently in each compartment (water, sediment) using an in-house script (Fig. S4), publicly available on GitHub (see Data Availability section). The pairwise comparisons were classified into four categories or data subsets:

(i) All pairwise comparisons between all samples (5,140 and 5,990 pairwise comparisons in sediment and water samples, respectively);
(ii) Intra-lake: pairwise comparisons between replicate samples of the same year and same lake (185 and 170 pairwise comparisons in sediment and water samples, respectively);
(iii) Inter-lakes: pairwise comparisons between samples of the same year in different lakes (“spatial variation”) (1,760 and 2,010 pairwise comparisons in sediment and water samples, respectively);
(iv) Inter-years: pairwise comparisons between samples of the same lake in different years (“temporal variation”) (510 and 600 pairwise comparisons in sediment and water samples, respectively).

The comparisons among replicates were not included in the “spatial variation” and “temporal variation” calculations. For each pairwise comparison between samples, we retrieved the βNTI and RCBray indices, along with the assigned assembly process, as output. The pairwise comparisons were grouped per “type of comparison,” which includes the four possible combinations of pairwise comparisons between replicates A and B from a given year and lake, as shown in Fig. S4. The number of occurrences of each assembly process was summed per type of comparison and converted to a percentage representing the relative proportion of each process in a given type of comparison. The average proportion (or relative contribution) of each assembly process and its standard deviation were calculated among all comparison types within each data subset. Significant changes in assembly process contributions between two data subsets (e.g., inter-lake versus intra-lake, inter-lake versus inter-years) were assessed through an exact two-sided permutation test using the *perm.test* function in the exactRankTests R package (72) with 1000 permutations (Fig. S4).

## RESULTS

### Segregation of bacterial communities between sediment and water habitats

**Fig 2:**
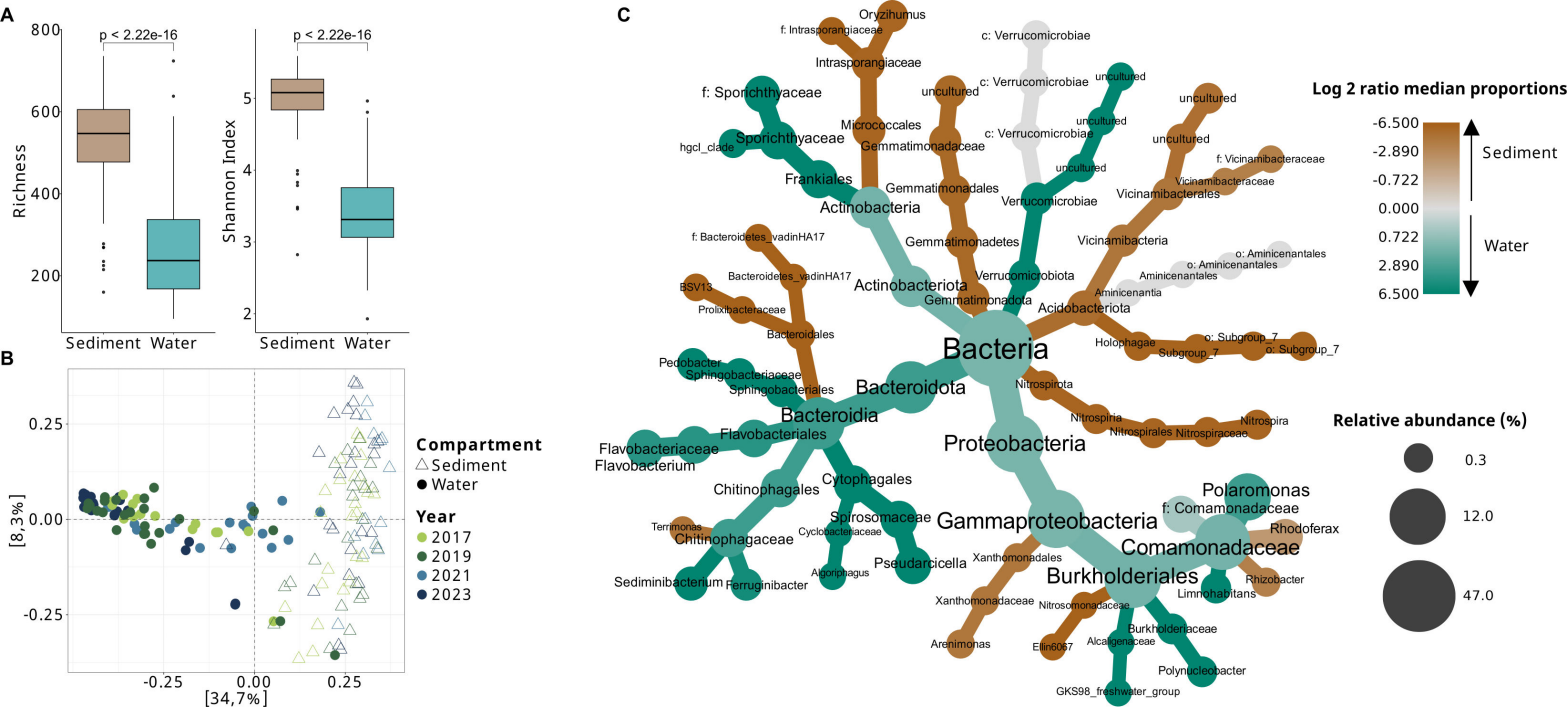
Analysis of alpha and beta diversity. (**A**) OTU richness and Shannon diversity index by habitat. The significant difference between habitats was evaluated using the Wilcoxon test. (**B**) Principal coordinate analysis (PCoA) based on Bray–Curtis dissimilarity after Hellinger transformation. Symbol shapes represent compartments, while colors indicate the sampling year. (**C**) Heat tree of 20 highest-scoring OTUs identified by linear discriminant analysis (LDA) for each habitat. The size of each node is proportional to the sum of relative abundance (with respect to the total data set) of the OTUs, summed per taxon when they shared the same taxonomic affiliation. The log2 ratio of median proportions between habitats was calculated to indicate taxa that differ in abundance between habitats. Taxa colored in brown are more abundant in the sediment, whereas those in turquoise are more abundant in the water habitat. The Wilcoxon rank-sum test was used to assess differences in median abundances between samples in each habitat. The list of discriminant OTUs is provided in Table S4, and their relative abundance is shown in Fig. S5.

The OTU richness and Shannon diversity index were significantly higher in surface sediment than in surface water samples (*P*-values < 0.001, Fig. 2A). The Principal Coordinates Analysis (PCoA) based on Bray–Curtis distances revealed that bacterial communities clustered primarily according to the habitat (sediment vs water), with separation along the x-axis explaining 34.7% of the variation in community composition (Fig. 2B). PERMANOVA confirmed that habitat was the primary driver of community composition differences (*R*² = 0.27, *P* = 0.001), followed by the lake identity (*R*² = 0.14, *P* = 0.001), whereas sampling year contributed only marginally to the observed variability (*R*² = 0.02, *P* = 0.001). The predominance of spatial (lake identity) over temporal (inter-annual) effects was consistent when analyzing surface sediment and water habitats separately (Table 1). The extent of community variability was not significantly different between water and sediment (betadisper, *P*-values = 0.92).

**TABLE 1:** Evaluation of the effects of habitat (i.e., water/sediment), sampling year, and ecosystem (i.e., lake identity) on the similarity of bacterial communities^*a*^

Variable	All samples		Sediment		Water	
	*R* ^2^	*P*	*R* ^2^	*P*	*R* ^2^	*P*
Habitat	0.27	0.001**	–	–	–	–
Year	0.02	0.001***	0.06	0.001***	0.03	0.004**
Ecosystem	0.14	0.001***	0.31	0.001***	0.36	0.001***

^*a*^ PERMANOVA test based on the Bray–Curtis dissimilarity index. Statistical significance is indicated as follows: ***P ≤ 0.001; **P ≤ 0.01. – indicates that no PERMANOVA test was performed in these subsets because the habitat effect could only be evaluated in the whole dataset.

Several OTUs were differentially abundant between habitats (Table S4; Fig. 2C). The 20 OTUs with the highest LDA scores in surface water primarily belonged to two main taxonomic groups: *Burkholderiales* (including the genera *Polaromonas*, *Limnohabitans*, and *Polynucleobacter*) and *Bacteroidia* (including the genera *Pseudarcicella*, *Sediminibacterium*, *Flavovacterium*, and *Pedobacter*). Other water-discriminant OTUs included members of *Sporichthyaceae* and *Verrucomicrobiae*. In contrast, OTUs enriched in the superficial sediment belonged to more diverse taxonomic groups, such as *Comamonadaceae* (*Rhodoferax, Rhizobacter*), *Intraporangiaceae* (*Oryzihumus* and unclassified), *Acidobacteriota* (*Aminicenantales* and *Vicinamibacterales*), *Nitrospira*, *Arenominas*, *Bacteroidetes*_vadinHA17, and *Gemmatimonadaceae* (Fig. 2C; Fig. S5).

OTU1 (*Polaromonas*) was the most abundant OTU in the surface water habitat (representing 16.5 ± 7.8% of the community), present in all surface water samples, and also detected in several superficial sediment samples at lower abundance (3.2 ± 4.9% of the community) (Fig. S5). OTU2 (*Rhodoferax*) was also present in both habitats, representing 2.2 ± 3.7% of the community in water and 3.4 ± 3.1% of the community in superficial sediment. When the samples were clustered based on the abundance profiles of compartment-discriminant OTUs, separation by year or ecosystem was not observed; however, separation by habitat was evident, except for some water samples (from ponds P1 and P2), which clustered with sediment due to their high solid content and possible sediment resuspension during sampling, typical of very shallow ecosystems (Fig. S5).

### Extent of spatial and temporal variability of lake bacterial communities

**Fig 3:**
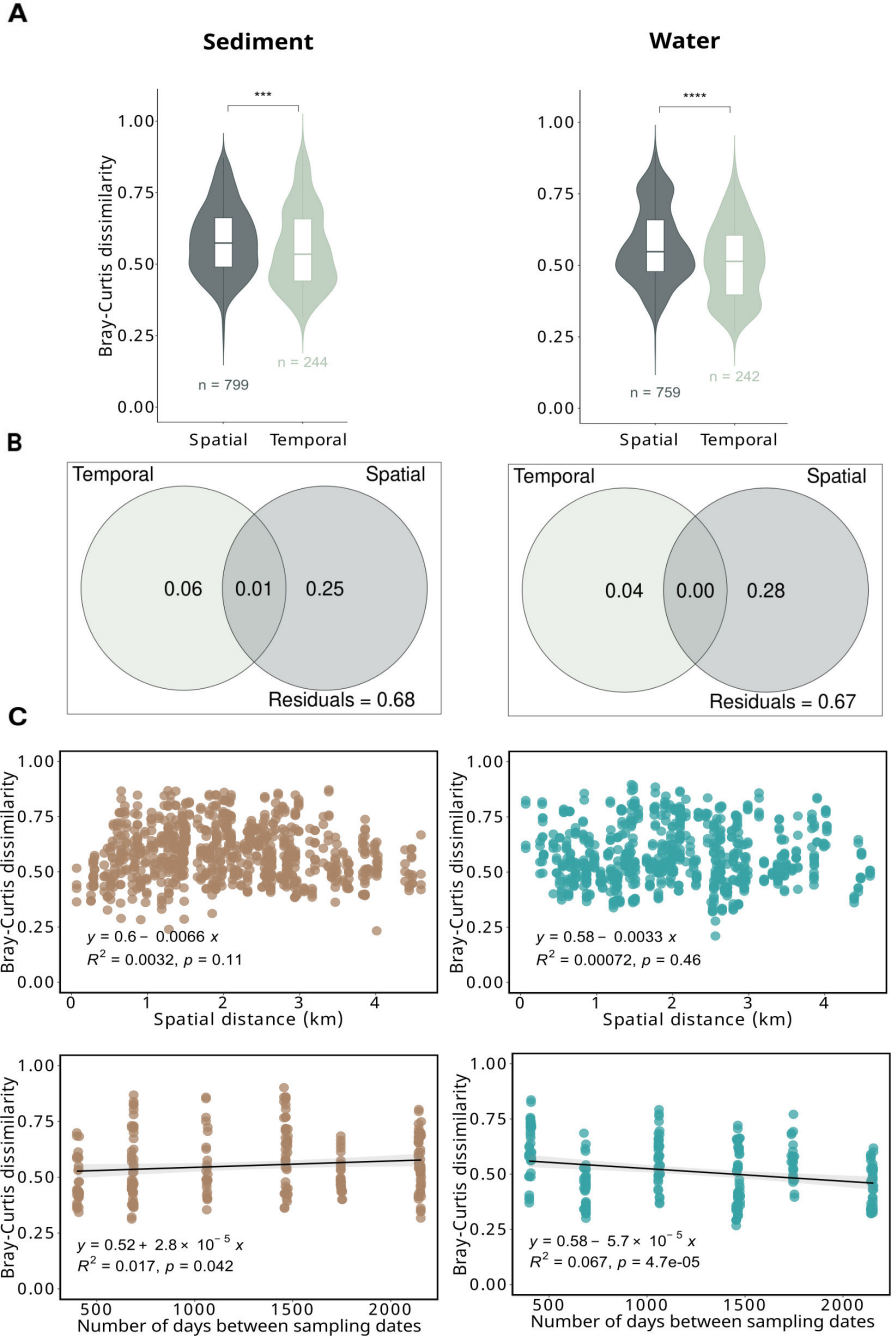
Spatial and temporal variability of bacterial communities. (**A**) Boxplot of Bray–Curtis dissimilarities showing the spatial variability (dissimilarity between pairs of samples from different lakes within the same year) and the temporal variability (dissimilarity between pairs of samples from different years within the same lake) for each habitat. Significant differences between spatial and temporal variations within each habitat were assessed by Wilcoxon tests (symbols indicating statistical significance are as follows: ***P < 0.001 and ****P < 0.0001). *n* represents the number of comparisons. (**B**) Variation partition analysis (VPA) based on Bray–Curtis dissimilarity matrices, showing the relative contributions of spatial and temporal factors on bacterial community dissimilarity. The significance of each factor was tested using 999 permutations, separately for each habitat (for each compartment, the significance was *P* < 0.05). (**C**) Distance-decay relationship (DDR) between spatial distance and community dissimilarity, and between temporal distance and community dissimilarity, for each habitat. The same pairwise comparisons as shown in panel (**A**) were used for each DDR. The temporal distance was calculated as the time elapsed between sampling dates.

To explore the spatial and inter-annual variation of the community structure, we compared the pairwise community dissimilarities across different ecosystems (i.e., spatial variation) and across different years (i.e., temporal variation) for each habitat independently. Spatial variation was significantly higher than temporal variation for both superficial sediment (*P* = 0.00048) and surface water (*P* < 0.0001) bacterial communities (Fig. 3A). The VPA confirmed that, in both habitats, a larger proportion of the variation in the bacterial community was explained by the spatial factor, which alone accounted for 25% of the variation in sediment and 28% in the water compartment, while the temporal factor explained to a lesser extent (6% for sediment and 4% for water compartment) (Fig. 3B).

We further investigated the extent of spatial and temporal variation of lake bacterial communities through the distance-decay relationship (DDR). At the geographical scale of the study, the dissimilarity of bacterial communities did not significantly increase with spatial distance (Fig. 3C, *P* = 0.11 for sediment and *P* = 0.46 for water). By contrast, the community dissimilarity decreased with temporal distance in the water compartment (*P* < 0.001), indicating convergence over time, while the community dissimilarity tended to increase over time in the sediments (*P* = 0.042), indicating divergence, albeit with low magnitude (*R*² = 0.07 and 0.02, respectively).

Interestingly, we observed a low inter-annual variability in surface water communities (lower than the spatial variability) despite high inter-annual variability of environmental parameters, which was even higher than their spatial environmental variation (Fig. S6A; all the measured physicochemical values are provided in Table S5). In other words, based on environmental parameters only, the different lakes clustered more by year than by ecosystem, as confirmed by PERMANOVA (Fig. S6B and C). This result gives even more weight to the relative temporal stability of water communities. When accounting for environmental factors in the VPA analysis of water communities, bacterial community variation was explained to a greater extent by spatial factors (24%) compared to environmental (6%) or temporal (3%) factors (Fig. S6B). The interaction between environmental and temporal factors accounted for only 1% of the variation in bacterial communities, while the interaction between environmental and spatial factors accounted for 4% of the variation. Although environmental changes occurred over time and space, their combined influence on bacterial community structure was very limited, especially over time. We cannot exclude that unmeasured physicochemical parameters could have a different impact on microbial communities.

### Conserved fractions of lake bacterial communities across time and space

**Fig 4:**
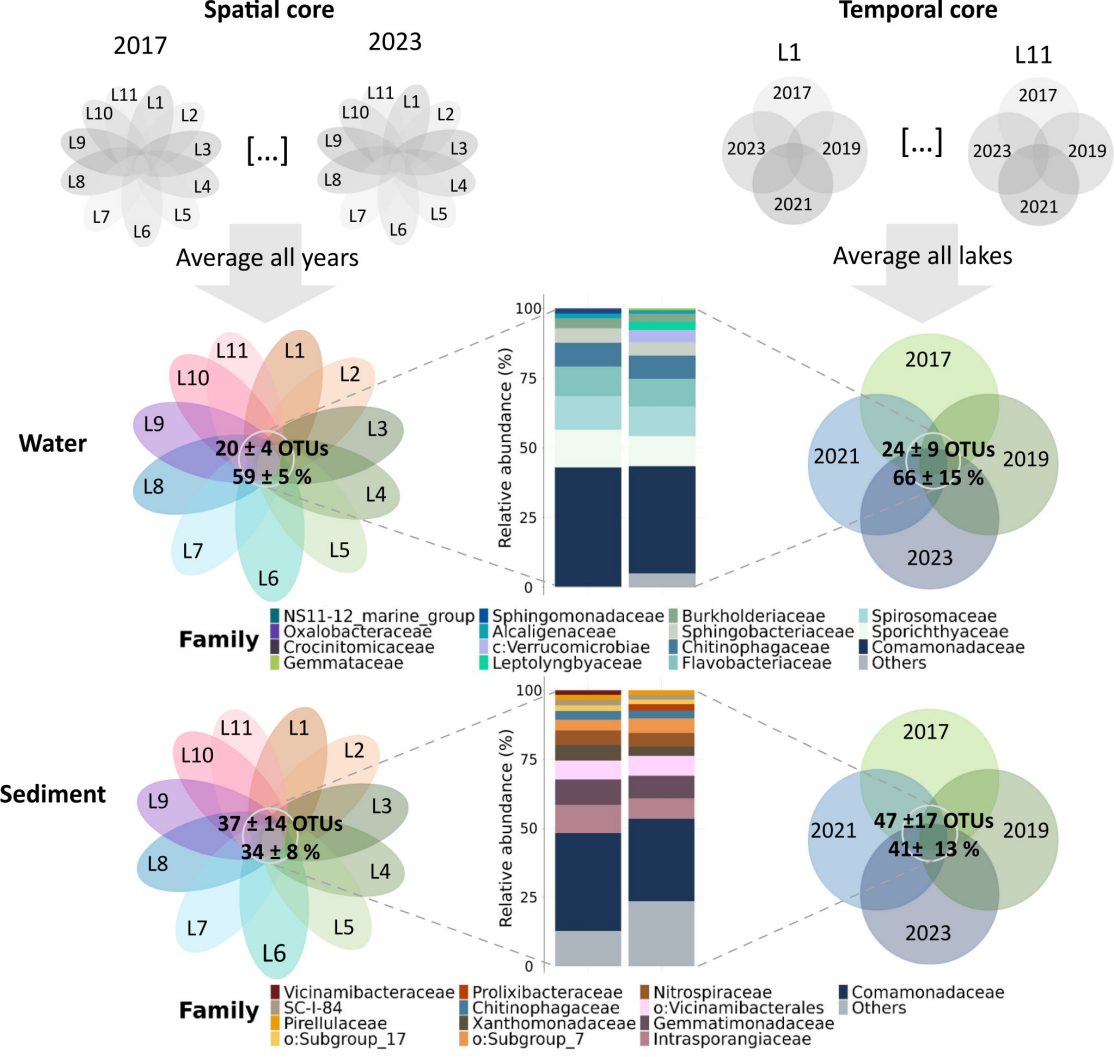
Spatial and temporal core microbiomes. The spatial core is composed of OTUs shared among 75% of the lakes for a given sampling year. The temporal core is composed of OTUs shared among 75% of the sampling years for a given lake. The detection level was set to 0.1%. The richness (OTU number) and relative abundance of the spatial and temporal cores are averaged among years and lakes, respectively, and the standard deviation is indicated. The relative core abundance was calculated relative to the total abundance in each year (for the spatial core) and in each lake (for the temporal core). The L1 to L11 terms represent a conceptual notation of the 11 studied ecosystems presented in Fig. 1. The average taxonomic composition of the spatial and temporal core microbiomes is presented next to each core, at the family level (or at the lowest taxonomic level available, indicated by the first letter before the taxonomic affiliation; o: order; c: class), showing the 12 most abundant families in each core. The common families in superficial sediment and surface water are represented by the same color. The detailed spatial cores for each year individually and the detailed temporal cores for each lake individually are provided in Tables S6 and S7 and Fig. S7.

The stability of bacterial communities between different years (temporal stability) and different lakes (spatial stability) was assessed by identifying and quantifying the temporal and spatial cores, respectively. In all cases, the cores represented important fractions of the total communities. In superficial sediment, the temporal core contained, on average, 47 ± 17 OTUs common to all years, whose cumulative relative abundance represented 41 ± 13% of the total community in each lake sediment, while the spatial core was smaller, containing 37 ± 14 OTUs common to all lakes, which represented 34 ± 8% of the total community in each year (Fig. 4). In the water compartment, the temporal core contained, on average, 24 ± 9 OTUs common to all years, whose cumulative relative abundance represented 66 ± 15% of the total community abundance in each lake. Here again, the spatial core was smaller, containing 20 ± 4 OTUs common to all lakes, representing 59 ± 5% of the total community abundance each year (Fig. 4). The higher abundance of temporal cores compared to spatial cores indicates that, in both habitats, bacterial communities are more stable over time than across space. These results are consistent with the pairwise community dissimilarities presented in Fig. 3A. The detailed spatial cores for each year individually and the detailed temporal cores for each lake individually are provided in Tables S6 and S7, and Fig. S7.

Strikingly, the 12 most abundant core families were highly similar between the temporal and spatial cores, indicating that the community fraction conserved over time was also conserved across different lakes (Fig. 4). The similarity of the temporal and spatial cores was also observed at the OTU level (Fig. S8). This result was consistent for both sediment and water habitats. The family *Comamonadaceae* was the most abundant in both spatial and temporal cores from both habitats. It was the only family common to surface water and superficial sediment cores at such a high abundance (>25%). The family *Chitinophagaceae* was also common to surface water and superficial sediment cores, but at a lower abundance (>2%). The other dominant taxa in the sediment spatial and temporal cores included *Intrasporangiaceae*, *Gemmatimonadaceae*, and *Acidobacteriota* members (*Vicinamibacterales*, *Vicinamibacteraceae*, and Subgroup_7 of the *Holophagae*) (Fig. 4). The *Prolixibacteraceae* family was specific to the temporal sediment core (Fig. 4). In surface water, the other dominant taxa (apart from the predominant water-sediment common taxa) of the spatial and temporal cores were *Sporichthyaceae*, *Spirosomaceae*, *Flavobacteriaceae*, and *Chitinophagaceae* (Fig. 4). *Leptolyngbyaceae* and *Verrucomicrobiae* were specific to the water temporal core (Fig. 4). For both water and sediment, some differences in core composition arose between individual lakes and between individual sampling years, such as a higher abundance of *Prolixibacteraceae* in the temporal cores of P1 and P2 sediment, or a higher abundance of *Leptolyngbyaceae* in temporal cores of P1 and P2 water, corresponding to pond ecosystems (Fig. S7).

### Assembly processes driving the spatial and inter-annual variability of the communities

**Fig 5:**
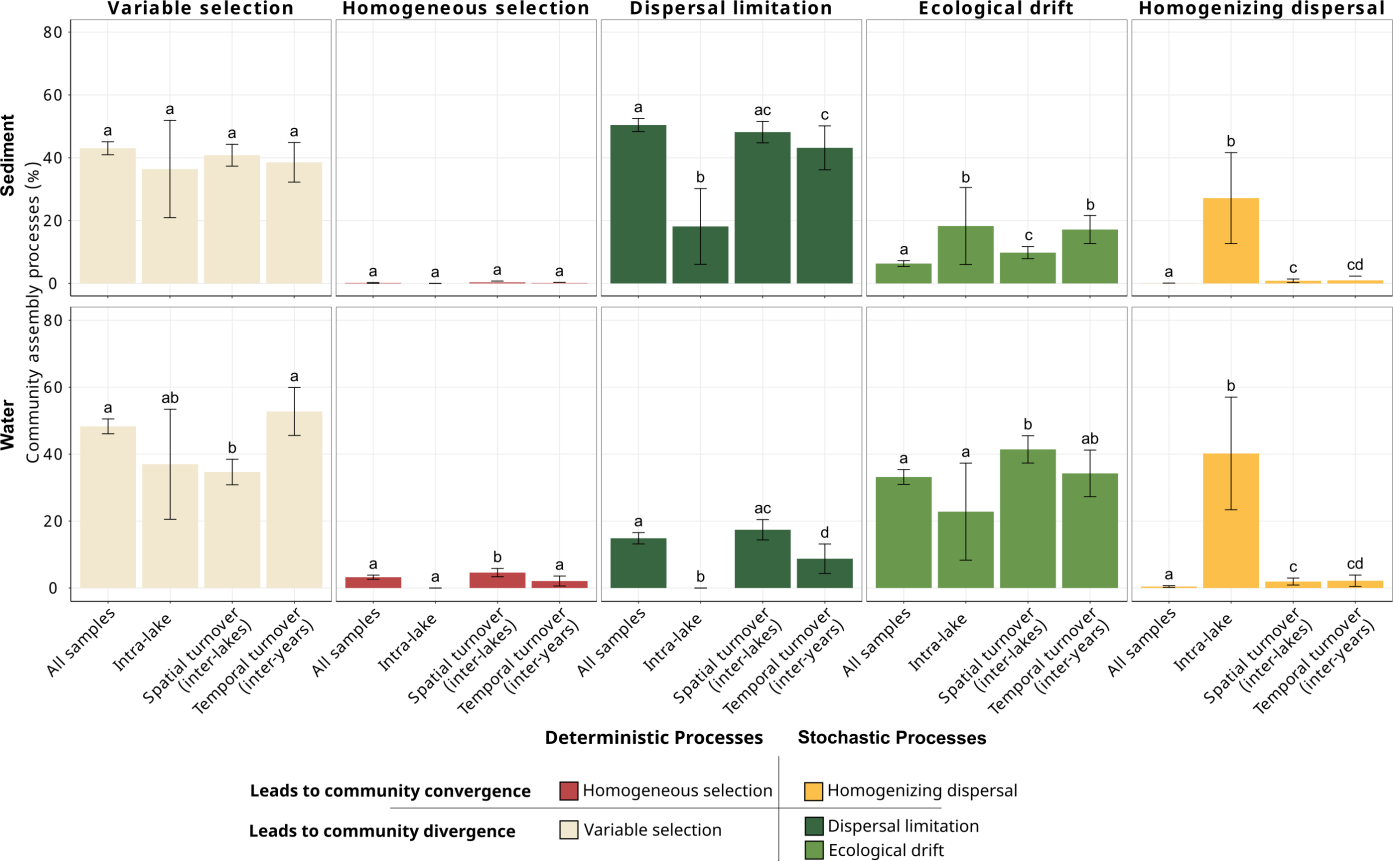
Quantification of bacterial community assembly processes. Mean relative contribution (%) of assembly processes for superficial sediment and surface water bacterial communities across different sample subsets: (i) all pairwise comparisons, including samples from different years and lakes; (ii) intra-lake scale, including samples from the same year and lake; (iii) inter-lake comparisons within the same year to assess spatial turnover; and (iv) inter-year comparisons within the same lake to assess temporal turnover. The proportions (or relative contributions) of each assembly process were averaged between all comparison types (see definition and detailed procedure in Fig. S4) within each data subset. Error bars represent the 95% confidence interval (CI), calculated from the standard error among all comparisons as detailed in Fig. S4. Different letters above the error bars indicate significant differences among sample subsets for a given habitat and assembly process, determined by permutation tests.

To gain insight into the ecological assembly processes underlying the inter-annual and spatial patterns of bacterial communities, we categorized the data set into four distinct groups of pairwise comparisons and present the mean values of assembly processes for each group. First, we examined the overall assembly processes across the entire data set, encompassing all possible pairwise comparisons (all samples, Fig. 5). In superficial sediment, assembly processes were dominated by stochastic processes representing 56.7% (with dispersal limitation and ecological drift contributing 50.4% and 6.3%, respectively), followed by variable selection (43%). Conversely, in surface water, assembly processes were evenly governed by deterministic processes (mainly variable selection, 48.3%) and stochastic processes reaching 47.9% (ecological drift and dispersal limitation representing 33.1% and 14.8%, respectively). Despite the low contribution of homogeneous selection, it was more prominent in water than in sediment.

This general pattern masks different behaviors at the intra-scale level (i.e., within a given lake at a given year) and along the spatial or temporal dimensions. When restricting the analysis to a smaller scale, comparing only replicates within the same lake and year, the contribution of homogenizing dispersal increased substantially, up to 27.2% in sediment, and became the predominant process in water (40.2%). Conversely, the contribution of dispersal limitation decreased compared to data sets encompassing different years and/or different lakes, consistent with the higher expected connectivity within lakes.

To elucidate the processes governing the spatial component of bacterial turnover, we focused on samples coming from different lakes during the same year, while the processes governing the temporal (inter-annual) component of bacterial turnover were obtained by comparing samples from different years within the same lake. In superficial sediment, the spatial and temporal turnovers of communities were governed by rather similar contributions of assembly processes, except a higher contribution of ecological drift explaining temporal variability than spatial variability (Fig. 5, *P* < 0.05). By contrast, in surface water, spatial and temporal turnovers of communities were governed by different contributions of assembly processes. For example, a higher contribution of variable selection and lower contributions of homogeneous selection and dispersal limitation explained temporal variability compared to spatial variability (Fig. 5, *P* < 0.05).

## DISCUSSION

### Distinctive bacterial communities between lake habitats

The most pronounced differentiation in bacterial community composition occurred between lake” habitats (i.e., superficial sediment vs surface water), regardless of variations across years and lakes. “Niche separation” (17) between planktonic and benthic lifestyle was a key driver of bacterial community assembly (73), despite the continuous nutrient, energy, and biomass exchange between sediment and the water column. The higher bacterial diversity in superficial sediments, consistent with previous studies (74–77), is likely due to the greater resource availability in sediments, the persistence of inactive/dormant bacteria (78, 79), and the sediment heterogeneity, which provides multiple available ecological niches, similar to patterns observed in soils (80–82). Several habitat-specific OTUs were identified. In surface water, the dominant OTUs (from *Polaromonas*, *Polynucleobacter*, and *Limnohabitans* genera) were consistent with previously reported taxa in Antarctic lakes, particularly in the Fildes Peninsula (43, 83, 84). *Polaromonas* is abundant on Arctic and Antarctic glacier surfaces and can be transported across large distances (85). Beyond these cosmopolitan clades, *Polynucleobacter* includes clades endemic to Antarctic lake water habitats (31). The genera *Flavobacterium* and *Pseudarcicella*, belonging to the phylum Bacteroidetes, also show predominance in water habitats in Antarctic lakes, consistent with our study (43, 84). Compared to water, studies on bacterial communities in Antarctic lake sediments remain limited. However, our findings are consistent with the identification of *Gemmatimonadota*, *Actinobacteria*, and *Acidobacteria* as dominant phyla in lake sediments from Byers Peninsula (Livingston Island, Maritime Antarctica) (86). The presence of *Polaromonas* in superficial sediment, although less abundant than in surface water, confirms its adaptability to diverse habitats, as previously reported in soils and cryoconite hole sediments (85, 87).

### Spatial variation dominates over inter-annual variation in shaping bacterial communities

Our results, based on Bray–Curtis distribution, PERMANOVA, VPA, and core microbiome analysis, showed that the spatial turnover (i.e., inter-lake variability, within a given year) was greater than the inter-annual turnover (i.e., inter-year variability, within a given lake) in both superficial sediment and surface water habitats. The spatial turnover can be attributed to several factors, including local factors (within habitat) such as abiotic conditions and biotic interactions, as well as regional factors operating at larger scales, such as geographic distance, dispersal, habitat isolation, connectivity, and history, with the importance of each factor varying significantly across large geographical gradients (25, 45). Here, DDR analysis showed that community dissimilarity did not increase with spatial distance, which may be due to the low spatial scale covered in our study. Water communities tended to converge with time, while sediment communities tended to diverge, likely reflecting the long-term integrative nature of sediments and their low connectivity, which leads to the gradual accumulation of changes. Despite this temporal variation in dissimilarity across years, spatial—rather than temporal—variation remained the dominant driver of community turnover. Moreover, here spatial distance had much more influence than environmental variation on water community dissimilarity, contrary to previous observations in temperate ponds (88). Indeed, for the objective of our study, we selected 11 lakes within a restricted area (~5.8 km²), with similar climatological and geological context (52), mostly of glacier origin (89), with sediments originating from basaltic bedrock weathering (90). Most of the lakes are connected by water streams, surface runoffs, and/or temporary gullies, either permanent or temporarily, especially during snow and glacier melting (89, 91, 92). By minimizing the environmental variations and heterogeneity between lakes, our objective was to focus on spatial variations between pseudo-replicate systems. On the other hand, the temporal (inter-annual) turnover integrates time-related variations of community, as well as environmental and/or climatic variations among years, along with the accumulation of past seasonal variability over the years (93). A notable finding of our study is the low inter-annual turnover of bacterial communities (lower than the spatial turnover), despite the inter-annual variability of environmental parameters (higher than their spatial variability) (Fig. S6). Even though assessing the environmental variability of the lakes was not the main scope of this work, our observation that environmental variables were not important drivers of microbial community composition might be due to the fact that the direction and significance of observed relationships between individual environmental parameters and microbial phyla varied from year to year as previously reported (94).

Studies comparing the relative impact of temporal and spatial variation with spatial and temporal (pseudo) replicates are scarce, and when they do, they often consider an intra-annual (seasonal) scale (95–97). Moreover, these studies mainly focus on different locations of specific ecosystems, such as soils (96–98), rivers (95), or anthropogenically impacted habitats (99). When they focus on lakes, these studies are often limited to a single lake (34, 41) or only target eukaryotic communities (44, 98). When considering seasonal fluctuations, the temporal variability usually exceeds the spatial variability (98, 99). This can be explained by the higher magnitude of intra-annual (seasonal) community turnover compared to inter-annual variations (98), as shown in lake bacterial communities (30, 31). Annually repeating patterns of microbial communities have been reported in lake (34) and in the ocean (100). Consistent with our results, spatial factors have been reported as more important drivers of microbial community than seasonal and inter-annual factors in soils across different spatial scales (96, 101), in subtropical river estuary (102–104), temperate freshwater ponds (88), and rainforest tree canopy (105). The hierarchization of spatial and temporal turnover also depends on the considered outcome variable. For instance, a stronger effect of spatial than inter-annual scale was reported in wetland soil community structure, but the opposite was observed at the functional trait level (106).

The proportion of persistent (core) community members across space and time was high (representing 59–66% of the community in water and 34–41% in sediment, respectively), contrary to a previous report in small freshwater temperate ecosystems at a comparably low (2–9 km) geographical scale (88), pointing to a high stability in our Antarctic ecosystems. Interestingly, the temporal and spatial core microbiomes exhibited highly similar taxonomic composition, indicating that the stable taxa over time are the same as those across space. This similarity not only indicates that the ecosystems have similar ecological filtering or contain effective dispersers, such as *Polaromonas* within the abundant *Comamonadaceae* family, but also suggests resistance and/or resilience of core taxa to seasonal environmental fluctuations. Consistently, a high persistence of taxa, such as *Polynucleobacter*, has been observed throughout the seasonal dynamics in a freshwater lake (31). Particularly, the temporal variation of physicochemical parameters does not induce a taxonomic change in the temporal core that differentiates it from the spatial core, indicating high stability of the communities in the face of environmental changes. The presence of certain taxa not shared among the cores may be associated with particular lakes due to specific abiotic conditions. For example, *Leptolyngbyaceae*, a cyanobacterium family present only in the temporal water core of shallow ponds P1 and P2 and not in their spatial core, has been identified as associated with microbial mats in Antarctic aquatic habitats (84).

### Bacterial assembly processes differ between water and sediment habitats

When we focused on the dominant processes by habitat (all-samples comparisons), we observed that approximately 50% of bacterial community assembly in both surface water and superficial sediment was governed by deterministic processes, primarily through variable selection (environmental filtering). The predominance of variable selection has been commonly observed in sediment bacterial communities, while stochastic processes are usually more important in water communities (32, 33, 35, 107). In our study, the type of stochastic processes largely varied depending on the habitat considered. Dispersal limitation was far more pronounced in sediments, consistent with previous findings (28, 47, 108). Our spatial core analysis also supported this pattern, suggesting more constrained dispersal of superficial sediment bacterial communities compared to those in surface water. This is expected, as the water habitat is a fluid system with high connectivity that facilitates bacterial dispersal, unlike the sediment (109). Additionally, sediments are inherently less exposed to external dispersal vectors such as wind or animals due to the “protective” effect of the water column. In the studied area, the lakes often remain physically isolated for extended periods because of seasonal ice cover. These factors can affect the potential for migration and immigration of bacterial communities (110).

Interestingly, ecological drift played a greater role in structuring bacterial communities in the surface water compartment. The high environmental variability of the surface water, which is more susceptible to perturbations, may reduce the consistency of selection pressures, thereby amplifying stochastic processes (15, 109). This aligns with a study showing that high fluctuations in fluidic ecosystems enhance ecological drift, particularly pronounced in small ecosystems such as some of our lakes (111). However, the literature remains ambiguous about the factors affecting ecological drift in different habitats. For example, our result can appear contradictory with expectations that ecological drift dominates in environments with limited spatial dispersal or in less dynamic environments (like sediment), and under weak selection pressure (20, 24, 45).

### Different contributions of assembly processes shaping the inter-annual versus spatial turnover of bacterial communities

In the superficial sediment habitat, the assembly processes explaining the spatial and inter-annual turnover are globally statistically similar between each other and with the all-samples comparison, characterized by a strong dominance of dispersal limitation and variable selection. The similar contribution of variable selection suggests a similar strength of environmental filtering over time and space. The strong contribution of dispersal limitation over time was comparable to that observed across space. The isolated nature of superficial sediments, affecting the potential for bacterial migration, could consistently restrict dispersal processes both spatially and temporally, as proposed by Soininen et al. (112). The only significant difference in the assembly process across spatial and temporal scales in sediment was a higher contribution of ecological drift explaining the temporal turnover compared to the spatial turnover. This pattern may also arise from restricted dispersal over time, which may amplify the influence of demographic stochasticity—random birth, death, and reproduction events—on community composition, resulting in greater ecological drift over time.

By contrast, in surface water, the inter-annual and spatial turnover were driven by distinct underlying assembly processes. Also, the deterministic variable selection process played a greater role in explaining temporal variation than spatial variation in the water compartment. This result suggests that environmental filtering of water communities over time (i.e., imposed by inter-annual environmental fluctuations) was greater than environmental filtering of water communities across space (i.e., imposed by inter-lake environmental differences). This is consistent with the fact that temporal variability of environmental parameters was higher than their spatial environmental variation. Temporal variation in lakes often reflects shifts in deterministic processes driven by changing environmental conditions, such as seasonal dynamics (29, 92), akin to spatial environmental heterogeneity across space. It has also been observed in other habitats such as rock pools (113, 114). As expected, dispersal limitation played a greater role in explaining spatial variation than temporal variation in water. Indeed, dispersal limitation is typically associated with spatial factors, where reduced connectivity or greater distances between sites limit dispersal rates (22, 41), which may explain the observed result.

### Possible limitations of the study

Our results are based on community characterization at the OTU level defined at 97% of similarity. However, the relative influence of the community assembly processes has been shown to vary with the phylogenetic resolution level (29). For instance, in Antarctic wetland communities, selection processes imposed stronger influence at finer (100% sequence similarity ASV) than at coarser (99%–97% sequence similarity OTUs) resolution (29). Moreover, our approach at the whole community level may hinder taxa-specific processes that could differ between phylogenetic clades, depending on their ecological strategies to thrive in extreme environments (48). Therefore, the absence of detected selection processes at coarser phylogenetic resolution does not imply their absence at finer resolutions. Future studies should assess community assembly processes at multiple phylogenetic resolutions and within different phylogenetic clades to improve our understanding of bacterial succession and colonization. When reprocessing our data at the ASV level, the main patterns (i.e., habitat segregation, stronger spatial than temporal effect demonstrated through PERMANOVA and VPA) were conserved, with the same magnitude, highlighting the robustness of the analysis (Table S8). Even if the scope of this study was to assess the inter-annual variability, we cannot exclude that shorter-term variability and/or local anthropogenic disturbances (115) could have an impact on the results. Future studies should increase the temporal resolution of sampling to take into account the potential for rapid changes in microbial communities over time. Finally, our study was limited to littoral samples, and we cannot ensure that the same conclusions would be drawn if samples were taken from different locations within the lakes.

### Conclusion

This study provides valuable insights into the structure and dynamics of bacterial communities in Antarctic lakes, emphasizing the importance of considering both spatial and temporal dimensions in order to get a full picture of bacterial variability. Our findings reveal that spatial variation had a greater influence on community composition than inter-annual variation in both surface water and superficial sediment habitats. Each habitat harbored a conserved core microbiome that persisted across sampling times, indicating high inter-annual stability and a certain degree of resilience toward possible (unmeasured) seasonal fluctuations that could occur between the inter-annual sampling dates. Deterministic assembly processes, dominated by variable selection, were prevalent in both habitats, but stochastic processes—such as dispersal limitation and ecological drift—differed markedly between habitats. Different assembly processes drive the inter-annual and spatial turnover of bacterial communities, with relative contributions modulated by the habitat type. These findings underscore the complex interplay between deterministic and stochastic processes in structuring bacterial communities and highlight how habitat modulates the influence of spatial and temporal drivers. Overall, this study emphasizes the importance of integrating both spatial and temporal dimensions when predicting bacterial responses to environmental changes in Antarctica and ecosystem shifts under global change scenarios.

## Data Availability

The FASTQ files of water and sediment bacterial communities were deposited in the Sequence Read Archive (SRA) database of the National Center for Biotechnology Information (NCBI) under the project number PRJNA1266680 with accession numbers ranging from SRX28894907 to SRX28895072 (see Table S2 for correspondence with sample nomenclature). The R codes used for ecological assembly process estimation in this study are publicly available on GitHub at https://github.com/DiegoAhumadaM/Ecological-Assembly-Processes-Estimation.

## SUPPLEMENTAL MATERIAL

Supplemental material aem.01079-25-s0001.pdf Tables S1 and S3 to S8; Fig. S1 to S8.

Table S2 aem.01079-25-s0002.xlsx Correspondence between sample nomenclature and NCBI codes.

ASM does not own the copyrights to Supplemental Material that may be linked to, or accessed through, an article. The authors have granted ASM a non-exclusive, world-wide license to publish the Supplemental Material files. Please contact the corresponding author directly for reuse.
