# Supplementary material for: Higher variability of bacterial communities across space than over time in Antarctic lakes, and contrasting assembly processes
Source: Appl Environ Microbiol. 2025 Oct 22;91(11):e01079-25. doi: 10.1128/aem.01079-25 (PMC12628676; doi:10.1128/aem.01079-25)
Supplement: Supplemental material — Tables S1 and S3 to S8; Fig. S1 to S8. [file aem.01079-25-s0001.pdf]

SUPPLEMENTARY MATERIAL FOR THE ARTICLE:

"Higher variability of bacterial communities across space than over time in Antarctic lakes, and contrasting assembly processes"

Diego Ahumada [a,b], Guillaume Schwob [b,c], Magdalena Osorio [b,d], Maria Soledad Astorga [e], Céline Lavergne [f,g], Nazli Olgun [h], Frederic Thalasso [i], Elie Poulin [a,b], Julieta Orlando [b,c], Léa Cabrol [b,j*]

***Corresponding author**

List of Supplementary Material:

Table S1. Study design.

Table S2: Correspondence of sample nomenclature used in this study and sample codes deposited on NCBI → see supplementary Excel File.

Table S3. Summary of sequence numbers per sample before and after pre-processing.

Table S4: List of the discriminant OTUs between water and sediment habitats.

Table S5: Physicochemical parameters measured in the water compartment of each lake across all sampling years.

Table S6: Spatial core microbiome.

Table S7: Temporal core microbiome.

Table S8: Community analysis at the ASV level.

Figure S1. Sampling periods and temporal variation in mean air temperature in Fildes Peninsula.

Figure S2. Rarefaction curves of species richness by sampling date and ecosystem.

Figure S3. Phylogenetic Mantel correlogram.

Figure S4. Assembly process calculation

Figure S5. Heat map representing the relative abundance of the 10 most discriminant OTUs from each habitat.

Figure S6. Evaluation of environmental variability and its contribution to bacterial community variation.

Figure S7. Community composition of the spatial core microbiome for each year independently, and of the temporal core microbiome for each lake independently.

Figure S8. Shared OTUs between temporal and spatial cores.

Table S1. Study design

Designation	Given name	Latitude	Longitude	Area (m^2)	Max. Depth (m)	Sampling year	Sampling date	Number of replicates per habitat	
								Sediment	Water
L1	-	-62.1948805	-58.9482333	2,452	< 1	2017	25-02-2017	2	1
						2019	10-01-2019	2	2
						2021	06-12-2021	2	2
						2023	20-01-2023	2	2
L2	-	-62.1912444	-58.9410166	2,805	< 1	2017	25-02-2017	1	1
						2019	12-01-2019	2	2
						2021	08-12-2021	2	2
						2023	20-01-2023	2	2
L3	-	-62.1893833	-58.9452667	3,272	< 1	2017	25-02-2017	2	1
						2019	14-01-2019	2	2
						2021	07-12-2021	2	2
						2023	16-01-2023	2	2
L4	Uruguay lake	-62.1865333	-58.9139333	70,933	16.5	2017	25-02-2017	0	1
						2019	14-01-2019	2	2
						2021	13-12-2021	2	2
						2023	16-01-2023	2	2
L5	-	-62.200885	-58.971292	6,417	9	2017	02-03-2017	2	1
						2019	09-01-2019	2	2
						2021	02-12-2021	0	2
						2023	12-01-2023	2	2
L6	Langer lake	-62.205801	-58.964396	23,001	6	2017	02-03-2017	2	1
						2019	09-01-2019	2	2
						2021	02-12-2021	0	2
						2023	12-01-2023	2	2
L7	Xi Hu Lake	-62.217496	-58.966291	11,888	4	2017	02-03-2017	2	1
						2019	09-01-2019	2	2
						2021	-	0	0
						2023	21-01-2023	2	2
L8	Yanou -	-62.220855	-58.958802	4,508	>1	2017	02-03-2017	2	1
						2019	09-01-2019	2	2
						2021	10-12-2021	2	2
						2023	23-01-2023	2	2
LK	Kitiesh Lake	-62.194899	-58.961742	93,527	11.5	2017	28-02-2017	8*	5*
						2019	17-01-2019	2	4*
						2021	09-12-2021	2	2
						2023	20-01-2023	4*	4*
P1	-	-62.1887667	-58.9245333	385	< 0.5	2017	25-02-2017	2	1
						2019	14-01-2019	2	2
						2021	13-12-2021	2	2
						2023	16-01-2023	2	2
P2	-	-62.2102929	-58.9407689	872	< 0.5	2017	03-03-2017	2	1
						2019	16-01-2019	2	2
						2021	-	0	0
						2023	13-01-2023	2	2

*In the Kitiesh lake, the sediment and water were collected from multiple sites within the lake, according to details given in Table S2. Values of 0 or 1 in the number of replicates per site indicate that it was not possible to collect or extract or sequence in duplicates.

Table S2: Correspondence of sample nomenclature used in this study and sample codes deposited on NCBI → see supplementary Excel File.

Table S3. Summary of sequence numbers per sample before and after pre-processing. The numbers are average ± standard deviation, calculated on the number of samples (N) per year.

Year	N sample	Number of sequences per sample before pre-process	Number of sequences per sample after pre-process	Number of OTUs per sample after filtering per abundance
2017	39	29,937 ± 7,011	17,733 ± 3,430	484 ± 147
2019	46	21,581 ± 5,567	13,271 ± 2,587	368 ± 169
2021	32	155,788 ± 47,639	105,541 ± 32,249	500 ± 178
2023	48	61,595 ± 12,782	42,845 ± 10,744	297 ± 162

Table S4: List of the discriminant OTUs between water and sediment habitats, identified by Linear Discriminant Analysis (LDA).

Habitat	OTU	LDA Score	*p-value*	*padj*
Sediment	OTU 14	-4.34	1.94E-18	1.94E-18
	OTU 09	-4.32	2.97E-17	2.97E-17
	OTU 22	-4.22	1.55E-26	1.55E-26
	OTU 02	-4.08	3.26E-05	3.26E-05
	OTU 21	-4.00	5.56E-15	5.56E-15
	OTU 28	-3.96	2.24E-15	2.24E-15
	OTU 23	-3.95	1.11E-17	1.11E-17
	OTU 50	-3.89	5.70E-13	5.70E-13
	OTU 27	-3.89	1.76E-18	1.76E-18
	OTU 38	-3.85	4.E-02	4.E-02
	OTU 32	-3.85	1.12E-18	1.12E-18
	OTU 35	-3.84	3.77E-13	3.77E-13
	OTU 56	-3.81	2.57E-19	2.57E-19
	OTU 40	-3.80	2.78E-19	2.78E-19
	OTU 48	-3.80	2.28E-10	2.28E-10
	OTU 24	-3.73	2.34E-07	2.34E-07
	OTU 62	-3.72	3.55E-20	3.55E-20
	OTU 51	-3.71	8.54E-21	8.54E-21
	OTU 92	-3.71	1.84E-22	1.84E-22
	OTU 25	-3.71	2.87E-07	2.87E-07
Water	OTU 01	5.13	8.28E-23	8.28E-23
	OTU 03	4.85	1.62E-22	1.62E-22
	OTU 06	4.74	1.74E-21	1.74E-21
	OTU 04	4.62	4.23E-20	4.23E-20
	OTU 07	4.54	3.61E-22	3.61E-22
	OTU 11	4.48	1.33E-25	1.33E-25
	OTU 20	4.33	3.11E-21	3.11E-21
	OTU 18	4.31	1.E-03	1.E-03
	OTU 15	4.29	5.77E-22	5.77E-22
	OTU 12	4.27	2.70E-19	2.70E-19
	OTU 34	4.23	2.00E-12	2.00E-12
	OTU 05	4.19	2.64E-16	2.64E-16
	OTU 44	4.15	5.23E-22	5.23E-22
	OTU 13	4.07	4.55E-23	4.55E-23
	OTU 30	4.06	7.37E-08	7.37E-08
	OTU 29	4.03	2.08E-08	2.08E-08
	OTU 17	4.03	3.27E-10	3.27E-10
	OTU 46	3.96	4.16E-24	4.16E-24
	OTU 52	3.91	1.16E-25	1.16E-25
	OTU 41	3.91	1.06E-10	1.06E-10

Table S5: Physicochemical parameters measured in the water compartment of each lake across all sampling years. In the Kitiesh lake, the values are averaged among multiple sites within the lake. ORP: oxidoreduction potential; DO: dissolved oxygen.

Ecosystem	Year	pH	ORP (mV)	DO (%)	Conductivity (µS/cm)	Turbidity (FNU)	Temperature (°C)	Organic Matter (%)
L1	2017	8.44	NA	93	100	NA	5.21	25
L2	2017	9.18	NA	100	37	NA	3.28	18
L3	2017	8.8	NA	100	68	NA	5.51	22
L4	2017	8.2	NA	87	95	NA	5.25	100
L5	2017	8.5	NA	97	115	NA	0.88	40
L6	2017	8.03	NA	90	206	NA	1.74	27
L7	2017	7.82	NA	84	75	NA	2.01	19
L8	2017	7.95	NA	97	111	NA	1.51	47
Lk	2017	7.4	NA	89	208	NA	2.52	NA
LK	2017	7.4	NA	89	208	NA	2.52	45
LK	2017	7.4	NA	89	208	NA	2.52	14
P1	2017	8.56	NA	98	57	NA	5.14	12
P2	2017	8.48	NA	100	256	NA	0.97	8
LK	2017	11.6	NA	101	176	NA	5.19	32
LK	2017	11.6	NA	101	176	NA	5.19	18
LK	2017	11.6	NA	101	176	NA	5.19	25
LK	2017	11.6	NA	101	176	NA	5.19	18
L1	2019	9	127.6	104.3	84	NA	4.88	12
L2	2019	8.01	116.5	76.3	200	NA	3.94	20
L3	2019	8.85	120	96.2	78	NA	3.37	17
L4	2019	9.23	121.6	100.6	122	NA	4.82	33
L5	2019	8.22	172.8	96	117	NA	3.95	36
L6	2019	9.44	141.8	112.5	313	NA	8.90	19
L7	2019	9.9	140.1	98.4	129	NA	5.13	18
L8	2019	9.18	145	99.5	103	NA	7.10	22
LK	2019	11.6	NA	12.2	176	NA	5.19	32
LK	2019	11.6	NA	12.2	176	NA	5.19	18
LK	2019	11.6	NA	12.2	176	NA	5.19	25
LK	2019	11.6	NA	12.2	176	NA	5.19	18
P1	2019	9.47	125.4	95.9	82	NA	2.56	9
P2	2019	9.39	103.8	NA	112	NA	9.23	10
L1	2021	6.94	217.3	90.3	102	1.1	1.27	NA
L2	2021	7.16	257	95.2	95.5	7.15	0.92	NA
L3	2021	6.82	327	90	96	5.5	1.56	NA
L4	2021	7.68	291.4	98.75	101	1.5	1.20	NA
L5	2021	7.22	209.5	83.7	99	1.2	0.77	NA
L6	2021	7.75	252.2	99.5	64	1.5	0.63	NA
L8	2021	7.95	316.9	107.35	101.5	1.35	1.05	NA
LK	2021	7.66	251.7	110.9	128	2.7	1.54	NA
P1	2021	7.41	329.7	92	48	48.3	0.78	NA
L1	2023	6.1	261	NA	103	13.2	5.33	19.1
L2	2023	7.35	223.6	NA	98	3.2	5.57	26.25
L3	2023	6.73	212.9	NA	86	31.4	7.21	21.77
L4	2023	6.03	204.5	NA	371	11.5	6.04	38.3
L5	2023	7.34	250.3	79.7	125	0.7	5.82	34.1
L6	2023	6.22	218.5	83.4	214	2.6	5.98	43.14
L7	2023	6.52	265.2	NA	103	1.1	6.26	41.46
L8	2023	8.15	208	NA	451	2	6.50	49.18
LK	2023	7.5	217	75	174	0.5	6.75	18.42
LK	2023	7.11	226.9	89.2	136	5.2	5.86	67.74

Table S6: Spatial Core Microbiome. Number of shared OTUs across lakes, computed for each year independently, with a detection threshold of 0.1% and prevalence in > 75% of the lakes. The relative abundance of the core is given in parenthesis and was calculated relative to the total community abundance in each year.

	Spatial core richness and abundance	
	**Sediment**	**Water**
2017	35 (34.5%)	26 (64.7%)
2019	35 (33.1%)	18 (57.0%)
2021	57 (43.0%)	17 (52.7%)
2023	22 (24.1%)	19 (61.2%)
Mean all years	37 (34 ± 8%)	20 (59 ± 5%)

Table S7: Temporal Core Microbiome. Number of shared OTUs across sampling years computed for each lake independently, with a detection threshold of 0.1% and prevalence in > 75%. The relative abundance of the core is given in parenthesis and was calculated relative to the total community abundance in each lake.

	Temporal core richness and abundance	
	**Sediment**	**Water**
L1	51 (41.4%)	20 (68.6%)
L2	63 (48.5%)	15 (52.8%)
L3	41 (35.0%)	15 (57.1%)
L4	64 (50.0%)	37 (87.1%)
L5	33 (43.3%)	17 (67.1%)
L6	27 (21.6%)	19 (45.7%)
L7	54 (41.6%)	30 (78.0%)
L8	55 (40%)	19 (72.8%)
LK	19 (18%)	44 (82.6%)
P1	46 (51.2%)	25 (41.4%)
P2	74 (65.2%)	26 (68.0%)
Mean all lake	47 (41 ± 13%)	24 (66 ± 15%)

Table S8. Community analysis at the ASV level. (A) PERMANOVA analysis at the ASV level. (B) Variance partitioning analysis (VPA) at the ASV level. The methodology of the analysis at the ASV level is provided in footnote [a] and the interpretation is provided in footnote [b].

A.

	All samples		Sediment		Water	
**Variable**	R^2	*p*	R^2	*p*	R^2	*p*
Habitat	0.17	0.001 ***	-	-	-	-
Year	0.02	0.004 **	0.03	0.001 ***	0.04	0.004 **
Ecosystem	0.16	0.001 ***	0.28	0.001 ***	0.39	0.001 ***

B.

	Sediment	Water
**Variable**	R^2	R^2
Temporal	0.03	0.04
Spatial	0.21	0.28

[a] Raw paired-end Illumina reads were processed using the DADA2 pipeline (Callahan et al., 2016). First, primers were removed with Cutadapt. Reads were filtered and truncated, removing low-quality bases and short sequences (parameters truncLen = 378, maxEE = 2, truncQ = 2 and minLen = 371) and sequencing errors were modeled to infer amplicon sequence variants (ASVs). Chimeras were identified and removed to obtain the final ASV table. Taxonomic assignment was performed using the SILVA reference database (v138.1). The resulting ASV table, taxonomy, and sample metadata were combined into a phyloseq object for downstream analysis. Reference sequences were stored and ASVs were renamed sequentially. Non-target sequences (chloroplasts, mitochondria, and eukaryotes) were removed. ASVs with extremely low abundance (relative abundance <0.005%) were filtered out, and samples with poor sequencing quality were pruned.

[b] The ASV-based PERMANOVA results show a higher effect of different lakes than the different years on microbial communities, with R2 coefficients highly similar to those obtained in the OTU-base PERMANOVA, thus confirming the robustness of our analysis. The ASV-based VPA results show a higher effect spatial than temporal variability to explain microbial variation, with R2 coefficients highly similar to those obtained in the OTU-based VPA, thus confirming the robustness of our analysis.

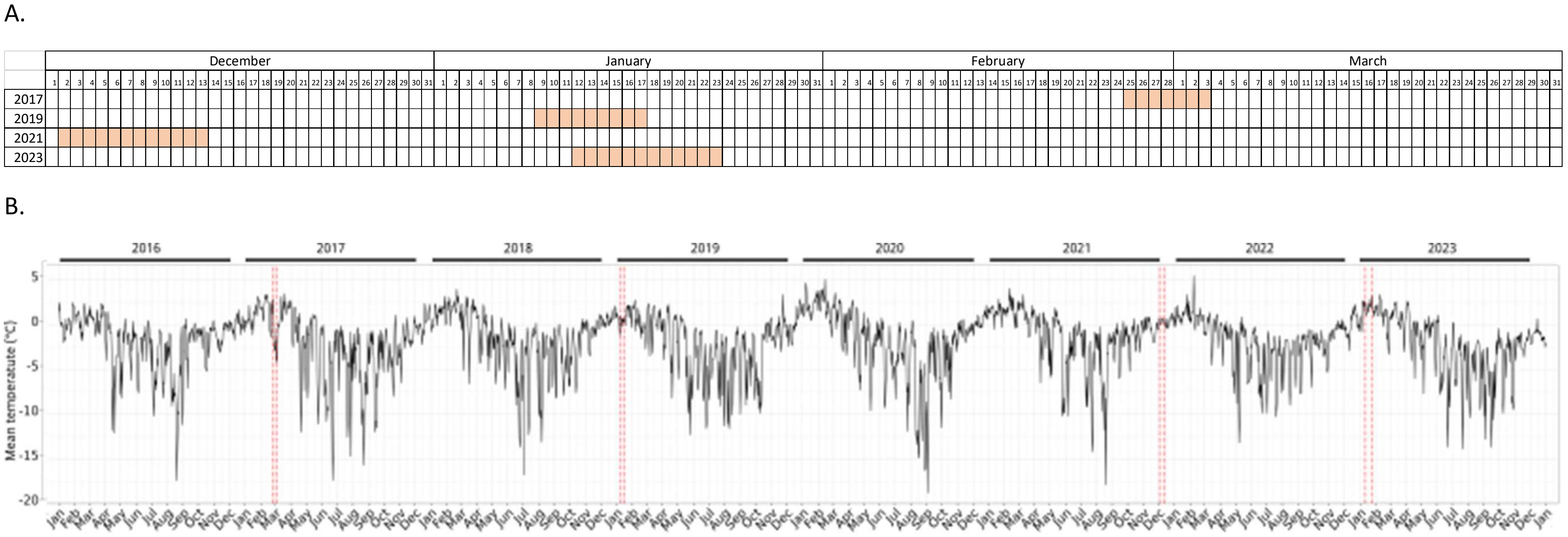

Figure S1. Sampling periods and temporal variation in mean air temperature in Fildes Peninsula. A. The sampling days are indicated in color for each year. **B.** The daily average of air temperature between 2016 and 2023 is shown, obtained from records of the Eduardo Frei Montalva Station. Red dotted lines indicate the time points at which the four sampling campaigns were conducted.

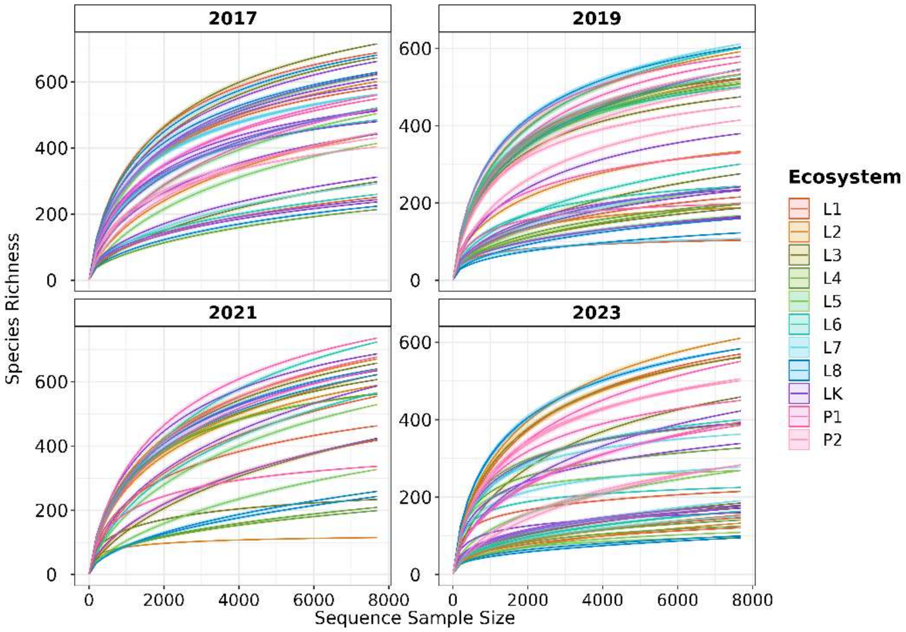

Figure S2. Rarefaction curves of species richness by sampling date and ecosystem. The curves are color-coded to represent the different ecosystems. The OTU table was rarefied to 7690 reads per sample after eliminating two sample with low abundance (L1-A sediment 2021 and LK-B sediment 2017).

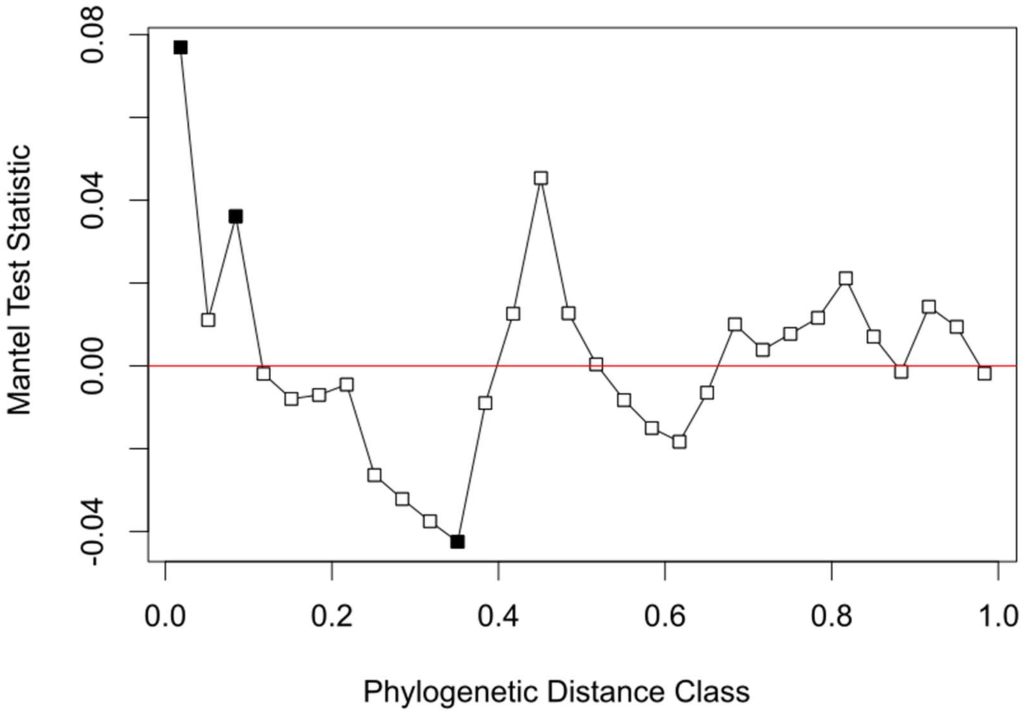

Figure S3. Phylogenetic Mantel correlogram. Solid symbols denote significant Pearson correlations between phylogenetic and environmental distances, at $P < 0.05$. Significantly positive correlations across short phylogenetic distances indicate that closely related OTUs share environmental optima. Phylogenetic distance class was normalized to vary between 0 and 1

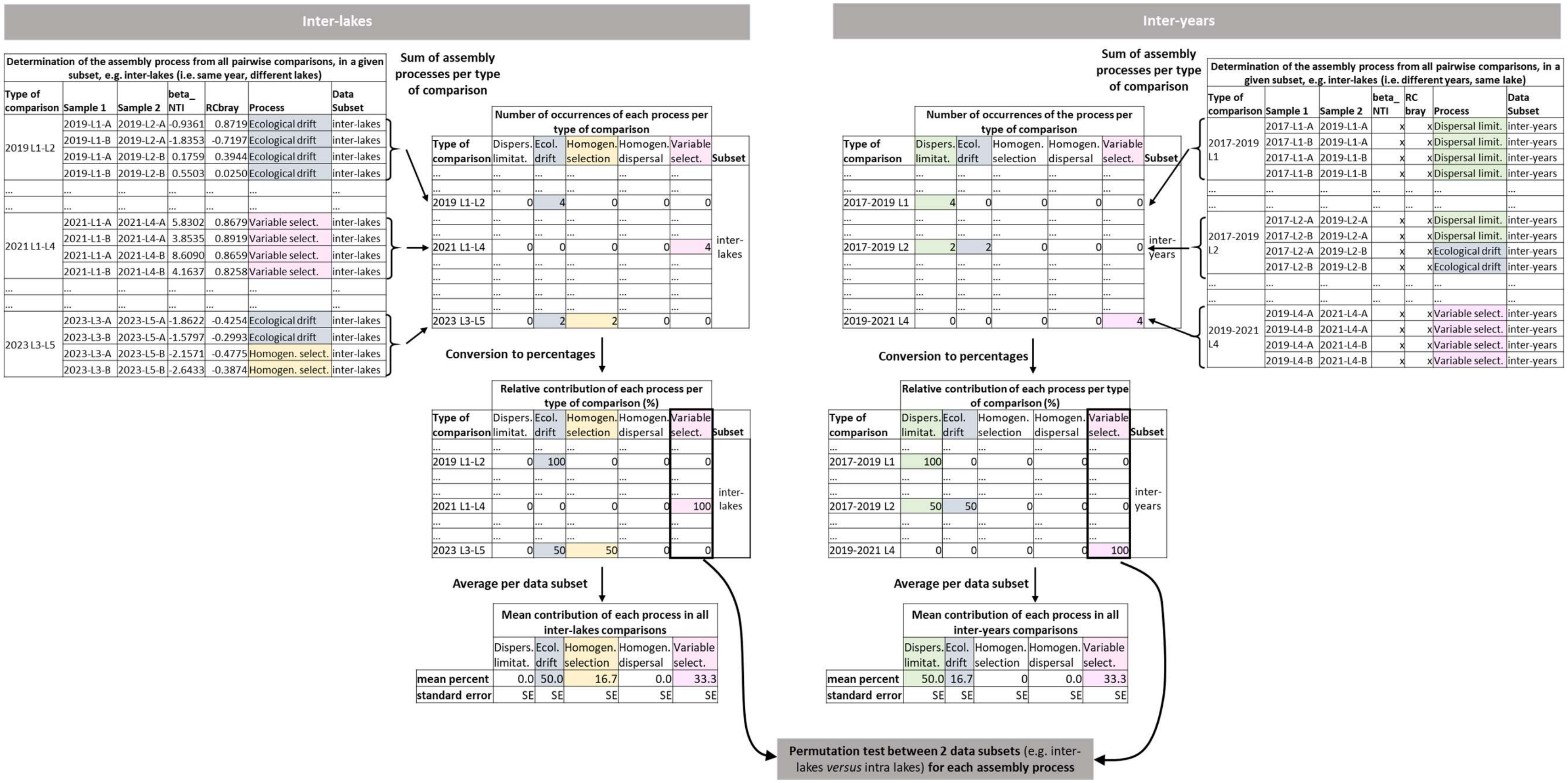

Figure S4. Assembly process calculation. Explanatory diagram showing the workflow used to calculate the relative contribution of the five community assembly processes in each data subset, and to test for significant changes in assembly processes contributions among data subsets. Each "comparison type" includes the four possible combinations of pairwise comparisons between replicates A and B from a given year and lake.

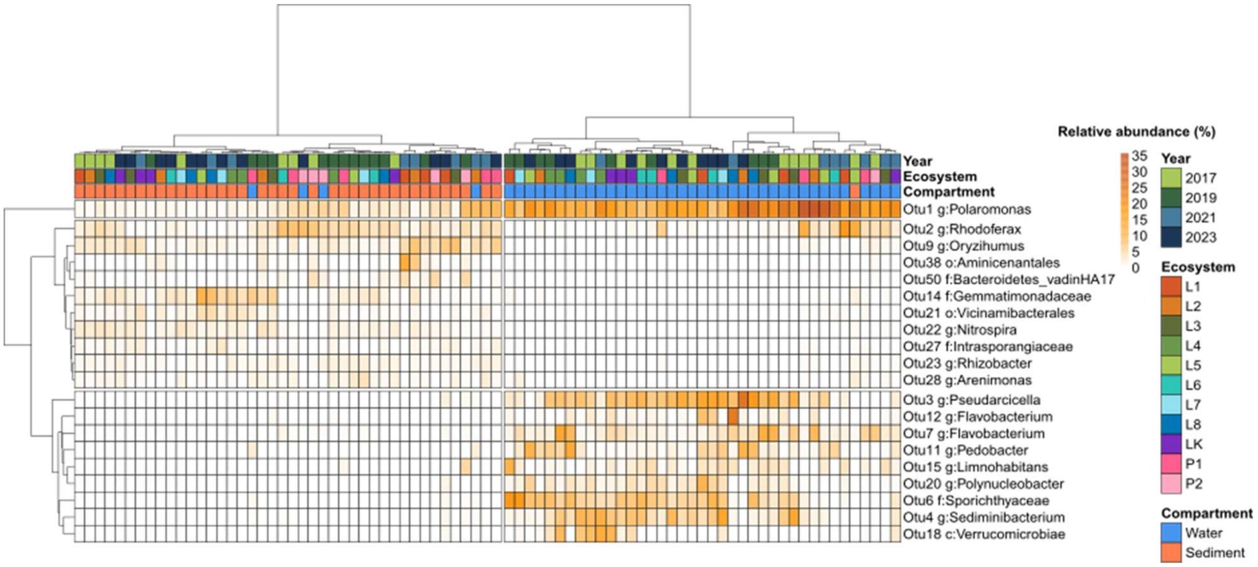

Figure S5. Heat map representing the relative abundance of the 10 most discriminant OTUs from each habitat. The samples are grouped according to a dendrogram constructed using Hierarchical Clustering with Ward's method. Rows were clustered according to the calculated Euclidean distance between OTUs based on their abundance across samples. The color scale indicates the OTU relative abundance per sample.

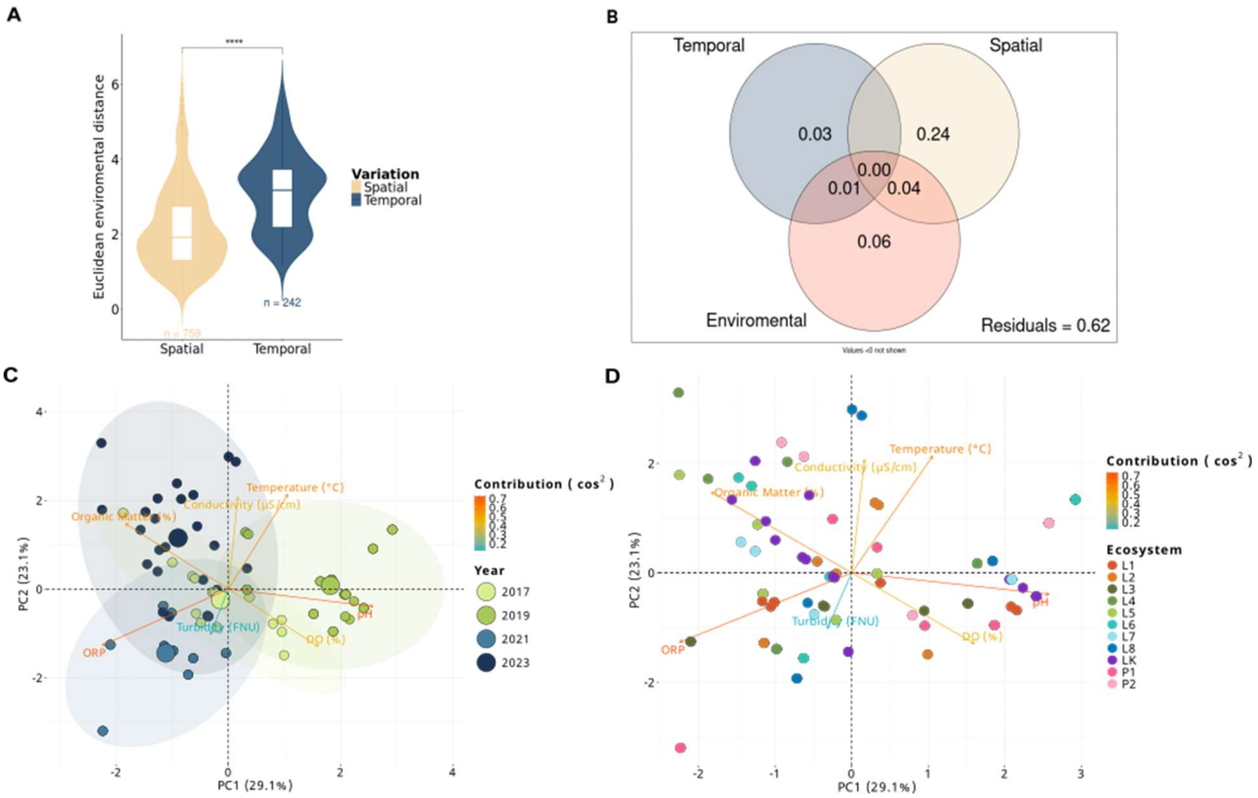

Figure S6. Evaluation of environmental variability and its contribution to bacterial community variation. (A) Boxplot of Euclidean environmental distance showing the spatial variability (distance between pairs of samples from different lakes within the same year) and the temporal variability (distance between pairs of samples from different years within the same lake) of environmental parameters, for each compartment. The environmental distance is calculated as an Euclidian distance from the matrix of forward-selected (with VIF < 10) environmental parameters. Significant differences between spatial and temporal variations within each compartment were assessed by Wilcoxon tests (The symbol indicating statistical significance corresponds to ****: $p < 0.0001$). n represents the number of comparisons (B) Variation partition analysis based on Bray-Curtis dissimilarity matrices, partitioning the relative contributions of spatial, environmental and temporal factors to bacterial community structure. (C, D) Principal Component Analysis (PCA) based on all environmental variables only, colored by year (C) or by lake (D). The environmental data set was normalized by using decostand (scales and center) function from vegan package and used to run a PCA (prcomp function). The arrows represent the environmental values and are coloured according to their contribution to the PCA based on the cos 2 . Based on environmental parameters only, the different lakes clustered more by year than by ecosystem. (PERMANOVA, Year $R^2 = 0.37$ $p = 0.001$; Ecosystem $R^2 = 0.17$ $p = 0.02$)

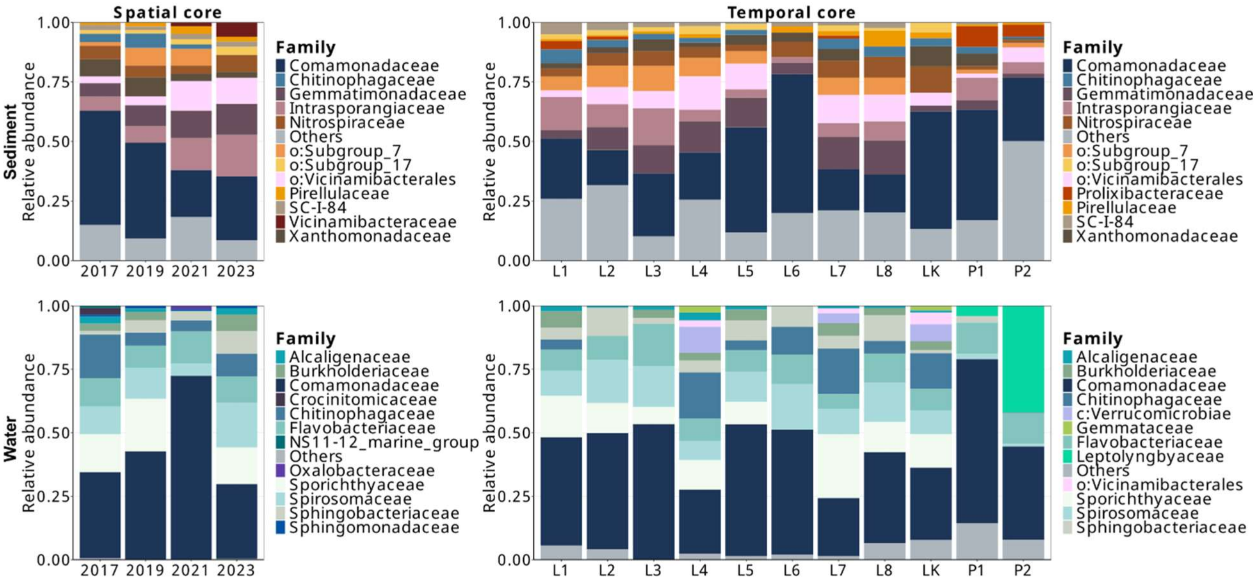

Figure S7. Community composition of the spatial core microbiome for each year independently, and of the temporal core microbiome for each lake independently. Relative abundance of the core microbial community across the ecosystems for each year (spatial core) and dissimilarity across the years in each ecosystem (temporal core), with relative abundances of the 12 most abundant Family taxa identified in the 'core' community.

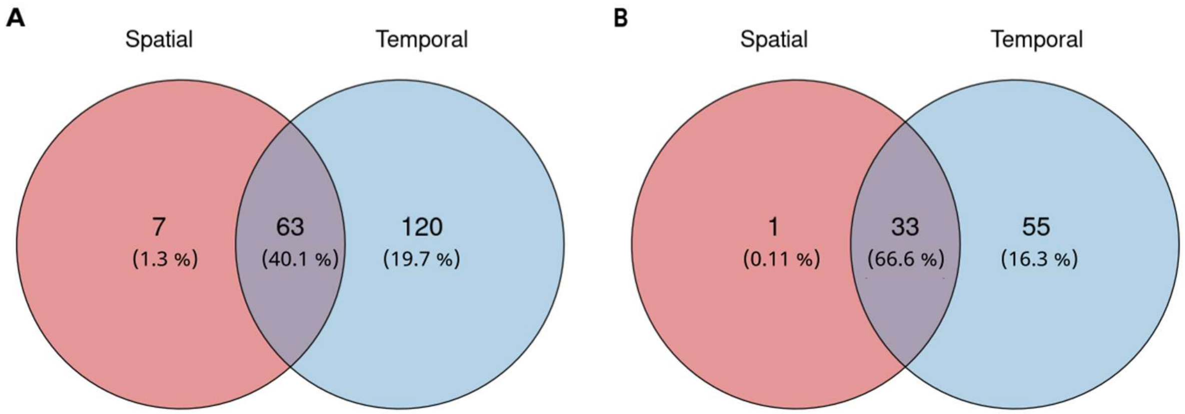

Figure S8. Shared OTUs between temporal and spatial cores, for sediment (A) and water (B) habitats. Frst we computed a global spatial core (including all the OTUs from the 4 year-specific spatial cores, with the function *unique*: giving 70 OTUs in sediment, 34 in water) and a global temporal core (including all the OTUs from the 11 lake-specific temporal cores, with the function *unique*, giving 183 OTUs in sediment, 88 in water). Then we looked for the common fraction between these global temporal and spatial cores (*ggvenn* function). In sediment: The results show that from the 70 and 183 OTUs of the spatial and temporal cores, 63 were shared. However, in terms of relative abundance, the shared OTUs represented 40.1 % of all sediment community, whereas the OTUs specific to the spatial and temporal cores represented only 1.3% and 19.7%, respectively. In water: from the 34 and 88 OTUs of the spatial and temporal cores, 33 were shared. However, in terms of relative abundance, the shared OTUs represented 66.6 % of all water community, whereas the OTUs specific to the spatial and temporal cores represented only 0.1% and 16.3% of the water, respectively. Therefore the spatial and temporal cores are composed of a majority of similar OTUs.
